# A Case of Traumatic Splenic Arteriovenous Fistula Treated by Transcatheter Arterial Embolization

**DOI:** 10.7759/cureus.63986

**Published:** 2024-07-06

**Authors:** Toshiro Imamoto, Makoto Sawano, Takahisa Hirano

**Affiliations:** 1 Department of Emergency Medicine and Critical Care, Saitama Medical Center, Saitama Medical University, Kawagoe, JPN

**Keywords:** splenectomy, coil, transcatheter arterial embolization, arteriovenous fistula, splenic injury

## Abstract

Transcatheter arterial embolization (TAE) has increasingly replaced surgery for treating solid organ injuries, including the spleen, due to its minimally invasive approach. Studies show only a 3% splenectomy rate after TAE, despite a 10% incidence of missed vascular injuries in the American Association for the Surgery of Trauma (AAST) grade III splenic injuries on initial computed tomography (CT) scans. However, there's a lack of high-quality studies recommending specific CT follow-up intervals after non-operative management (NOM) of splenic injuries or guidelines for initiating treatment in cases of pseudoaneurysms or arteriovenous fistulas (AVFs).

Here, we discuss the case of a 44-year-old man who presented with a splenic injury due to a motor vehicle accident. The splenic injury was AAST-spleen grade III, but because there was no evidence of extravascular leakage or AVF formation, NOM was selected. CT on the fifth day showed a pseudoaneurysm and an AVF, for which TAE was performed on the seventh day, preserving most of the parenchyma of the spleen with no complications.

The indications for NOM as a treatment strategy for splenic injury are expanding, but since the 2018 revision of the AAST grading, the grade changes depending on the presence or absence of vascular injury, but in some cases, it is difficult to determine the presence or absence of active bleeding by CT findings. In fact, it has been reported that more than 25% of vascular lesions do not show up on CT, although CT has good sensitivity in detecting active bleeding, and the rate of NOM failure is higher in AAST grade III and above, so early angiography is likely to be useful.

Splenic AVF may present with few symptoms in the early stages but may present with extrahepatic portal hypertension in the late stages, and patients may present to the hospital with symptoms such as abdominal pain and diarrhea. TAE is often the treatment of choice in traumatic cases, and the extent of embolization is important in the balance between preserving splenic function and completing treatment.

The shift towards conservative management of splenic trauma may increase the occurrence of splenic AVFs. Transcatheter coil embolization of segmental branches has been effective in treating posttraumatic splenic AVFs, preserving splenic immune function and reducing risks linked to surgery and splenectomy.

## Introduction

In the treatment of solid organ injuries, transcatheter arterial embolization (TAE) has come to partly replace surgical options because of its less invasive nature. The same is true in splenic injuries [1]. A study that evaluated the rate of spleen preservation and complications after TAE reported that only 3% of patients underwent splenectomy after TAE [2]. In addition, in the American Association for the Surgery of Trauma (AAST)-spleen grade III injuries, there is a 10% incidence of occult vascular injuries not identified by initial computed tomography (CT) at the time of presentation [2]. However, there are no high-quality studies that recommend specific CT follow-up intervals after choosing non-operative management (NOM) for splenic injuries or when to initiate treatment when a pseudoaneurysm or arteriovenous fistula (AVF) is present.

In this report, we present a case of splenic injury with multiple traumatic splenic AVFs, in which endovascular treatment was selected, and although the embolization area was extensive before treatment and the possibility of splenectomy was a concern, we were able to minimize the embolization area by TAE utilizing the coil characteristics and avoid splenectomy. We present the case with a review of the literature.

## Case presentation

The patient was a healthy 44-year-old man. He was injured when he struck a road sign while driving a motor vehicle late at night. He was transported to a hospital near the accident scene for initial treatment, where CT showed a sternal fracture, rib fracture, and splenic injury. The patient’s vital signs were stable when he arrived at the hospital, but his blood pressure gradually began to decrease, and he was transferred to our hospital due to difficulties in management. The patient arrived at our hospital five hours after the injury. His vital signs before transfer were: blood pressure (BP) 78/42 mmHg, pulse rate (PR) 71 beats/min, respiratory rate (RR) 18 breaths/min, saturation of percutaneous oxygen (SpO_2_) 100% (on ambient air), and body temperature (BT) 37°C. Prior to transport, the patient's hemodynamics were unstable. The patient arrived at our hospital after receiving six units of red blood cell (RBC) concentrate and four units of fresh frozen plasma (FFP). Vital signs on arrival were: BP 110/51 mmHg, PR 86 beats/min, RR 20 breaths/min, and SpO_2_ 99% (ambient air). Blood tests showed no coagulopathy or anemia (Table 1).

**Table 1:** Laboratory examination findings PaCO_2_: partial pressure of arterial carbon dioxide; PaO_2_: partial pressure of arterial oxygen; HCO_3_: bicarbonate; BE: base excess; Hb: hemoglobin; Plt: platelet; PT-INR: prothrombin time-international normalized ratio; APTT: activated partial thromboplastin time

Parameter	Patient Value	Reference Range
pH	7.393	7.35 to 7.45
PaCO_2 _(mmHg）	44.4	35 to 45
PaO_2 _(mmHg)	87	90 to 100
HCO_3 _(mmol/L)	37	22 to 26
BE (mmol/L)	2.1	-2.5 to +2.5
Lactate (mmol/L)	1.8	0.56 to 1.39
Hb (g/dL)	10.7	14 to 18
Plt (×1000/μL）	17.6	15 to 40
PT-INR	1.03	0.85 to 1.15
APTT (sec)	22.2	20 to 40
Fibrinogen (mg/dL)	212	180 to 320
D-dimer (μg/mL)	14.4	＜1

**Figure 1:**
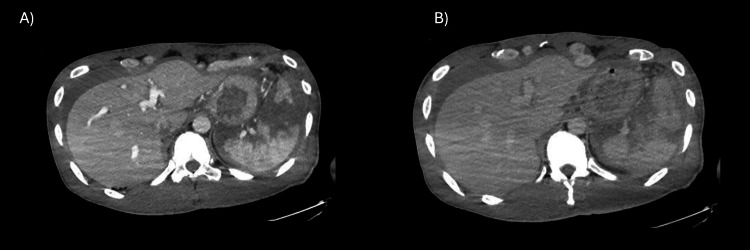
Enhanced whole-body CT axial view (A) arterial phase and (B) portal-venous phase Neither extravascular leakage images nor pseudoaneurysms are seen. CT: computed tomography

Contrast-enhanced CT was repeated at our hospital (Figure 1).

**Figure 2:**
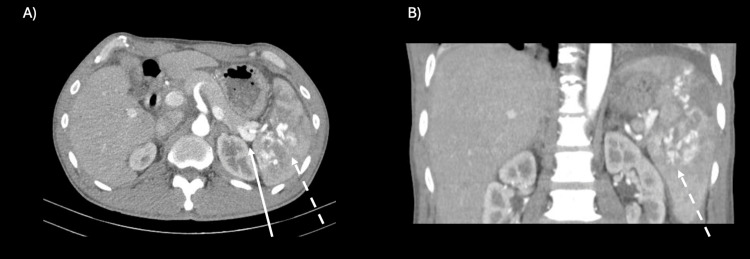
Contrast-enhanced CT on day five of hospitalization (A) shows splenic injury in horizontal view in the early phase of contrast-enhanced CT, with pseudoaneurysm (white dotted arrow) and early depiction of splenic vein (white arrow), suggesting a traumatic AVF via a pseudoaneurysm. In (B), the coronal view of the early phase of contrast-enhanced CT shows the same site as in (A), indicating multiple pseudoaneurysms (white dotted arrow). CT: computed tomography; AVF: arteriovenous fistula

The patient was judged to be AAST-spleen grade III with no evidence of extravascular leakage at that point, and NOM was selected. Since the patient’s vital signs did not deteriorate, a follow-up CT was performed on the fifth day. The CT showed multiple pseudoaneurysms, which were not seen on the first CT, and some early splenic veins were seen, suggesting a traumatic splenic AVF (Figure 2).

**Figure 3:**
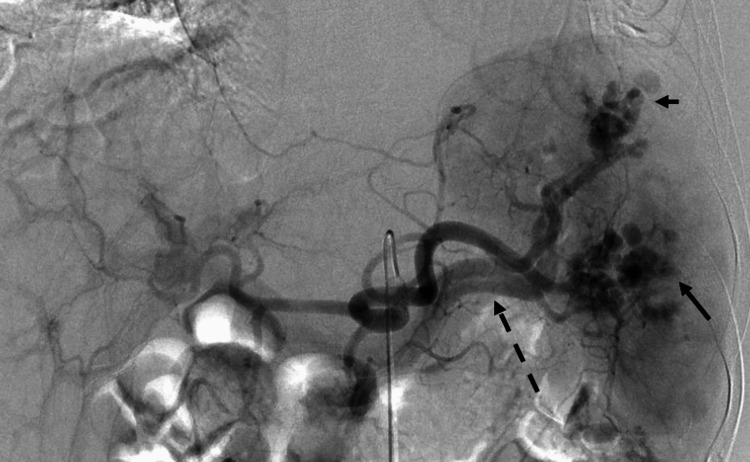
Angiography from the celiac artery (before TAE) There are multiple pseudoaneurysms (black arrow) and a concurrent AVF (black dotted arrow) in the spleen. TAE: transcatheter arterial embolization; AVF: arteriovenous fistula

Therefore, it was decided to perform TAE on the seventh day. Celiac arteriography showed multiple pseudoaneurysms and AVFs, as seen on CT (Figure 3).

The 4-Fr shepherd’s hook-type contrast catheter was replaced by a 4.5-Fr shepherd’s hook guiding sheath (Parent Plus 45, Medikit, Tokyo, Japan), with a 4-Fr guiding catheter (Cerulean G, Medikit, Tokyo, Japan) and a 2.9-Fr high-flow microcatheter (Carry Leon, UTM Co., Aichi, Japan). This system was designed to allow the microcatheter to reach the end of the more tortuous splenic artery, with the guiding sheath providing a firm backup in the aorta.

**Figure 4:**
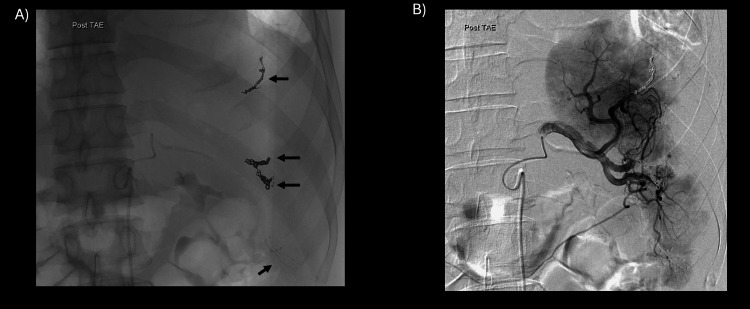
Final angiography from the splenic artery (after TAE) (A) There is a pseudoaneurysm and AVF at the end of the upper pole branch, one at the third branch branching off the main trunk of the upper pole, and two at the end of the lower pole. Three are embolized with coils (black arrow) and one is embolized with NBCA (black dotted arrow). (B) The AVF has disappeared, and the splenic parenchyma is well preserved. TAE: transcatheter arterial embolization; AVF: arteriovenous fistula; NBCA: n-butyl-2-cyanoacrylate

In addition, to create a triple coaxial system, a 2.0-Fr selective catheter (Carry Leon, UTM Co., Aichi, Japan) was used to identify pseudoaneurysms and shunt points. Coil embolization was performed with isolation of each site (Figure 4).

Embolization was performed using AZUR soft 3D coils (Terumo, Tokyo, Japan) and Embold fibered coils (Boston Scientific, Marlborough, USA) depending on the lesion site. The final contrast images showed that the AVF had disappeared. We had presumed that embolization of the vessels responsible for multiple pseudoaneurysms would leave only a small area of splenic parenchyma and that extensive splenic infarction would eventually lead to complications such as abscess formation, necessitating splenectomy. In fact, however, the splenic parenchyma was preserved better than estimated. Postoperative follow-up CT showed no residual pseudoaneurysms and no evidence of abscess formation, and the patient was discharged on the tenth day.

## Discussion

The Eastern Association for the Surgery of Trauma (EAST) guideline is the most frequently used treatment strategy for splenic injury, and we adhered to it; in 2018, the AAST splenic injury scale was modified to consider the presence of vascular injury [3]. This added that angiography should be considered in cases of (i) severe injury of AAST grade III or higher, (ii) extravascular leakage by contrast-enhanced CT, (iii) intra-abdominal bleeding of moderate severity or greater, and (iv) expected persistence of bleeding. There are also reports that AAST grade IV or higher injuries are associated with NOM failure, requiring strict management [4].

In the present case, the initial CT did not show extravascular leakage, but it did show more than moderate intra-abdominal hemorrhage. Additionally, the extent of ischemia in the splenic parenchyma could be judged to have exceeded 25% at this point, and upon retrospective review, it is possible that the AAST grade should have been determined to be IV instead of III.

If AAST grade IV had been used, there is a possibility that NOM would have failed, as described in the previous literature, and it could have been presumed that a pseudoaneurysm or splenic AVF in the subacute phase, as in the present case, would have been present and required TAE.

Since there was no extravascular leakage on CT at the initial presentation, it was determined that no active hemorrhage, pseudoaneurysm, or splenic AVF was present. However, since the extent of ischemia was more than 25%, it is presumed that the vessels within the spleen showed intimal injury and the parenchyma distal to it had become ischemic but recanalized in the subacute phase, forming multiple pseudoaneurysms and splenic AVF as seen in this case. It has been reported that CT has good sensitivity in detecting active hemorrhage, but more than 25% of vascular lesions are not detected [5]. It is possible that a small pseudoaneurysm would have been detected in this case if angiography had been performed on the first day of admission.

Splenic artery embolization is often performed as distal to the splenic portal as possible to reduce the risk of infarction while treating the vascular lesion. The choice of this embolization site is controversial, especially in the presence of multiple vascular lesions [6]. Generally, proximal splenic artery embolization (PSAE) is performed when multiple splenic parenchymal injuries are present, whereas distal splenic artery embolization (DSAE) is performed in cases of localized vascular injury. Considering this, PSAE could be selected in the present case.

Infarction of the splenic parenchyma occurs at a higher rate with DSAE; it has been reported that there is no significant difference in the splenic preservation rate between DSAE and PSAE [7]. In the present case too, the embolization of multiple vascular lesions could be extensive, and it was explained to the patient beforehand that there was a possibility of splenic necrosis and splenectomy could be required. However, it was possible to preserve about 40% of the splenic parenchyma by identifying a super-selective embolization site, using a soft coil with gel and a fibered coil with a high radial force, which allowed embolization in a short segment to prevent extensive embolization.

Regarding splenic function, there is a paucity of literature on the percentage of embolization that would result in loss of function. In partial splenic embolization, which is performed as a treatment for portal hypertension, the appropriate embolization percentage is said to be 50-70% of the entire spleen [8]. Basic research has shown the same view that approximately 30-50% preservation is required to maintain normal splenic function [9]. In other words, embolization of more than 70% would have a negative effect on splenic function.

The SPLEEN-IN study evaluated the rate of splenic sparing and complications in patients who underwent TAE for splenic injury. Complications were low at 5.6%. Splenectomy was required after TAE in 3% of patients. Interestingly, angiography in AAST grade III injuries showed 18 occult vascular injuries not identified on initial CT [2].

The problem is that there are no high-quality studies that recommend a specific CT follow-up interval after choosing NOM for splenic injuries or when to start treatment if a pseudoaneurysm or AVF is present. In previous reports, most NOM interruptions occurred within six to eight days of admission [10]. Haan et al. reported that only two of 140 patients with AAST grades I and II had worsening findings on CT follow-up one to two days after injury, but clinical symptoms were also worsening in both cases [11]. Some believe that CT follow-up is not necessary in all cases. On the other hand, Weinberg et al. reported that 11 of 330 patients with low-grade splenic injuries had pseudoaneurysms [12].

Considering that the accessibility to CT examination in Japan differs from that in Western countries, we believe that CT follow-up for blunt splenic injuries at around one week is appropriate.

In the present case, multiple pseudoaneurysms and an AVF were observed on a five-day follow-up CT and whether these aneurysms and AVF could resolve spontaneously was considered controversial.

At our institution, the treatment was initiated when the size was determined by CT to be treatable. Splenic AVFs are relatively rare, with a higher percentage of idiopathic than traumatic cases, and large AVFs can be seen [13]. In traumatic cases, TAE is the treatment modality about half the time, and endovascular treatment can often be completed, as in the present case [14]. When a splenic AVF is suspected, angiography is very useful because it allows dynamic image acquisition, although various modalities such as contrast-enhanced CT, magnetic resonance imaging (MRI), and color Doppler ultrasound are said to be useful [15].

Splenic AVFs do not cause any symptoms in the early stages, but they present with a variety of symptoms in the late stages. Specifically, they can cause abdominal pain, diarrhea, ascites, and hemorrhage. Extrahepatic portal hypertension can lead to cirrhosis, and transvenous embolization (TVE) may be required in cases of overlooked and marked dilatation [16].

We believe that early intervention is important for splenic AVF because it can cause serious late complications if left untreated, even if it is asymptomatic in the early stages. Possible complications of treatment include splenic infarction and abscess formation, but the development of catheters and coils has made super-selective embolization possible.

In recent years, there have been several reports of treatment of splenic AVF using coils and n-butyl-2-cyanoacrylate (NBCA) [17].

In the future, we would like to analyze these data using CT volumetry and determine specific cut-off values for the limits of splenic parenchymal preservation.

## Conclusions

There are splenic injuries that do not show extravascular leakage or AVF immediately after trauma, but they become apparent in the subacute phase. If TAE is technically feasible, aggressive TAE may be beneficial for such patients. Embolization should be performed to preserve as much parenchyma as possible, taking into consideration the characteristics of the coils used.
